# Socio-cultural barriers to the delivery and utilisation of child healthcare services in rural Ghana: a qualitative study

**DOI:** 10.1186/s12913-022-07660-9

**Published:** 2022-03-03

**Authors:** Felix Kwasi Nyande, Esmeralda Ricks, Margaret Williams, Sihaam Jardien-Baboo

**Affiliations:** 1 Department of Nursing, School of Nursing and Midwifery, University of Health and Allied Sciences, Ho, Ghana; 2 Department of Nursing Science, Faculty of Health Sciences, Nelson Mandela University, Port Elizabeth, South Africa

**Keywords:** Socio-cultural, Child healthcare, Utilisation, Barriers, Language barrier, Traditional beliefs, Self-medication

## Abstract

**Background:**

Over half of global deaths among children under five years of age occur in sub-Saharan Africa. Prompt and consistent access to and utilisation of child healthcare services improves child health outcomes. However, socio-cultural barriers impede the utilisation of child healthcare services among rural dwellers in Ghana. There is a paucity of studies that explore the experiences of nurses and caregivers regarding the socio-cultural barriers to the delivery and utilisation of child healthcare services in rural areas in Ghana such as the Nkwanta South Municipality.

**Purpose:**

The purpose of this study was to explore the experiences of nurses and caregivers regarding the socio-cultural barriers that impede the delivery and utilisation of child healthcare services by caregivers for their children in the Nkwanta South Municipality, Ghana.

**Methods:**

Data were collected through semi-structured interviews conducted with a purposive sample of ten nurses and nine caregivers of children under five years of age who utilised the available child healthcare services in a rural setting. The consent of all participants was sought and given before interviews were conducted. Data analysis entailed coding and the generation of themes the codes.

**Results:**

The exploration of experiences of nurses and caregivers of children under-five years of age revealed that certain socio-cultural beliefs and practices, language barriers and reliance of caregivers on self-medication were the main socio-cultural barriers that impeded the delivery and utilisation of child healthcare services in the Nkwanta South Municipality.

**Conclusion:**

Nurses and caregivers experienced several socio-cultural barriers which either delayed care seeking by caregivers for their sick children or interfered with the smooth and prompt delivery of needed child healthcare services by nurses. Some of the barriers negatively affected the interaction between nurses and caregivers with the tendency to affect subsequent child healthcare service utilisation. It is recommended that healthcare managers and nurses should foster close collaboration with caregivers and community leaders to address these socio-cultural barriers and facilitate prompt and consistent utilisation of child healthcare service in rural areas.

## Background

Child mortality has remained a global health concern and particularly in Sub-Saharan Africa which contributes more than half of the global deaths of children under five [1]. The risk of dying before the age of five is 12 times higher for a child born in Sub-Saharan Africa as compared to their counterparts born in high income countries [1]. Child health indicators measure the wellbeing of children; and these indicators are far from desirable in Ghana. At the end of 2015, Ghana failed to meet the Millennium Development Goal four on improving child health [2].

The utilisation of child healthcare services in Ghana has remained low in rural areas where socio-cultural beliefs and practices regarding healthcare and orthodox healthcare services utilisation are also very rife, which have contributed to the poor child healthcare outcomes of rural areas as compared to urban areas in Ghana [3, 4]. This situation could hamper the country’s attainment of the child health target of the Sustainable Development Goal three which aims to reduce neonatal mortality to at least as low as 12 deaths per 1,000 live births and under-five mortality to at least as low as 25 deaths per 1,000 live births, by the year 2030 [5].

According to the 2017 Ghana Maternal Health Survey, the infant and under-five mortality rates in Ghana were 37 and 52 deaths per 1000 live births respectively [6]. Most of the deaths of children under five are due to preventable or treatable causes like malaria, pneumonia, anaemia, and diarrhoeal diseases [7]. These diseases are more prevalent in the rural areas of Ghana, where poverty is also endemic [8, 9].

Prompt and consistent access to and utilisation of child healthcare services improves child health outcomes [10]. However, challenges such as the socio-cultural aspects of people’s ability to use health services impact their utilisation of these services [11]. The Three Delays Model provides a framework for examining the various causes of delay in maternal and child healthcare services utilisation that could contribute to adverse outcomes [12]. The model identifies delays in the decision to seek healthcare, arriving at the healthcare facility, and receiving appropriate care as significant contributors to maternal mortality [12]. The first delay, that is, the delay in the decision to seek care is shaped by factors such as the actors involved in the decision-making; the status of women; characteristics of the illness; distance from the health facility; costs in terms of finance and opportunity; previous experience with the healthcare system; and perceived quality of care [12]. This delay highlights the important role of socio-cultural factors in healthcare decision-making. It is thus important to explore the socio-cultural barriers that contribute to delays in the utilisation of child healthcare services.

Studies from around the world have found various socio-cultural factors which impact the utilisation of child healthcare services; for example, cultural competency, ease of communication, gender-induced affordability, avoidance of social stigma and labelling, cultural expectations [13]; language barriers, cultural taboos, stigmatisation [14]. Also, age of the child, recognition of danger signs [15]; acceptability of hospital care [16]; perception of orthodox health services, sense of tradition [17]; lack of respect, adherence to customs and influence of families and relatives [18] have been reported to influence the utilisation of healthcare services. Similarly, social factors including low maternal education, residing in a rural area, low family income, and high dependency including large family size, polygamy, and multiparity have been found to negatively influence child healthcare-seeking behaviour and service utilisation in Ghana [4]. These factors either facilitate or impede the utilisation of child healthcare services.

Again, in Sub-Saharan Africa, the socio-cultural barriers that impede the timely utilisation of child healthcare services have been extensively documented in the literature. Limitations on the social interactions of women and related gender dynamics; ignorance about the cause and treatment of a child’s illness and gender roles where men are the major decision-makers in children’s health impede the utilisation of child healthcare services [19, 20]. In a study by Lungu et al. [21], health service providers reported that caregivers of children under five delayed in seeking healthcare for their children’s illnesses because the caregivers resorted to traditional and alternative medicines as their first choice. According to Bosomprah et al. [22], the low importance mothers attach to immediate check-ups; ignorance of diseases of the newborn; and socio-cultural beliefs that allow babies to be outdoors only after the naming ceremony is performed negatively influenced utilisation of child healthcare services. Webster, et al. [23], also found that caregivers’ level of education and not the severity of the child’s condition influenced the choice of health facility, that is, health centres or hospitals, where the sick child is sent to. Although pneumonia is a major cause of child morbidity and mortality in Ghana, Abbey, and his colleagues [24], found that most rural caregivers in the Dangme West District in Ghana demonstrated very poor knowledge of the condition; they were thus unlikely to seek healthcare in cases of pneumonia in children. In another study that examined the effects of intra-familial decision making on women’s utilisation of maternal health services in Ghana, it was found that pregnant women were limited in their participation in the decision-making process when deciding whether and when to utilise these services as decisions were largely made by their husbands, mothers-in-law, or other community members, based on their beliefs and values [25]. This signifies the control that family members have over mothers and their children in terms of decision-making relating to the health-seeking behaviour of caregivers of children.

Globally, nurses constitute the largest group of the healthcare workforce and the backbone of primary healthcare service delivery [26]. In rural areas in Ghana, nurses play the crucial role of bridging the gap of essential healthcare workforce shortage at the primary level; a situation occasioned by the persistent disparities in the distribution of essential health workforce between urban and rural areas [7, 27]. Additionally, community Health Nurses, who are the community health officers, constitute the majority of the workforce ensuring the success of the Community-based Health Planning and Services (CHPS) policy [28, 29].

From the foregoing discourse, it is evident that socio-cultural barriers impede the delivery and utilisation of child healthcare services especially among marginalised populations such as rural dwellers. Rural dwellers are already faced with numerous barriers to the utilisation of healthcare services; hence these socio-cultural barriers worsen their situation. Again, most previous studies within the Ghanaian and other contexts elsewhere tend to focus mainly on the caregivers with a few focusing on the perspective of nurses; however, only a few of these studies have explored these socio-cultural barriers from the perspective of both nurses and caregivers.

There is however a paucity of literature relating to Ghana on the experiences of nurses and caregivers of children in rural areas regarding the socio-cultural barriers that impede the delivery and utilisation of child healthcare services. This study aimed to explore the experiences of nurses and caregivers regarding the socio-cultural barriers to the delivery and utilisation of child healthcare services in rural Ghana.

## Methods

### Design

The researchers conducted a qualitative study using an exploratory, descriptive, and contextual design [30]. A qualitative design aided the researchers to gain insights into the meaning and experiences regarding socio-cultural barriers to child healthcare services utilisation from the perspective of nurses and caregivers in a rural setting [31]. A contextual design entails the study of phenomena as they exist within a particular context [32]. A contextual study considers the geopolitical and socio-cultural circumstances of the setting and population of the study. Everyday real-life events are studied in the specific context in which they occur [33]. By using the contextual design, the researchers were able to explore the socio-cultural barriers to child healthcare service utilisation within a rural setting as this design is responsive and sensitive to the way of life of the population being studied.

### Study site

The study was conducted in both health facilities and communities within the Nkwanta South Municipality, located in the Oti Region of Ghana. The Nkwanta South Municipality is mostly rural with most of the residents engaged in peasant farming. About 75% of the population of the Nkwanta South Municipality live in rural areas [34]. The municipality is one of the most ethnically diverse districts in Ghana where about a quarter of all Ghanaian languages are spoken [35]. Also, nearly 41% of the population has never attended school. Over 80% of all those with some education in the municipality did not go beyond basic education [34, 36].

Malaria and anaemia are among the top causes of hospitalisation among children in this area [34]. The municipality has two hospitals, a health centre, four clinics, and several CHPS compounds that provide healthcare services to the surrounding communities [34].

### Selection of participants

The participants consisted of nurses and caregivers who were purposively selected with the goal of obtaining participants who may have experienced the phenomenon under study and can help answer the research questions [31]. The inclusion criteria for nurse participants were that they had to be a registered nurse or midwife; in full-time employment at the health facilities in the Nkwanta South Municipality; directly involved in the delivery of child healthcare services for no less than six months prior to the interviews. Six months was necessary to ensure that participants had adequate experience in the provision of child healthcare services to contribute to the study. Inclusion criteria for caregivers were that they must have had a child or be taking care of one that is less than five years of age; have utilised any child healthcare services in the municipality at least twice within the past 12 months; be a resident of the study area.

The nurse participants were sampled from the two hospitals in the municipality, a health centre, and two CHPS compounds. These facilities were purposively sampled to ensure adequate representation of nurses from the different levels of the Primary Healthcare system and different locations within the municipality. Following an introduction by the nurse manager and/or unit heads, nurses who worked at these facilities were approached and invited for participation by one of the authors who conducted the interviews. Communities of the caregivers were purposively sampled from within the catchment areas of the nurse participants in the study. With the assistance of community health nurses and community health volunteers within the health facilities, caregivers who met the criteria for inclusion in the study were identified and approached to participate in the study by the field investigator (FKN) who conducted all the interviews. The interviewer is fluent in *Twi* language, so there were no challenges with caregivers who could only express themselves in *Twi* language. All participants approached by the researcher agreed to take part in the study. All the nurse participants and six of the caregivers who met the inclusion criteria were selected from health facilities. The remaining three caregivers were selected from different communities within the municipality. Nurse unit managers acted as ‘gatekeepers’ for nurses and caregivers who were recruited from their units within the health facilities. The sample size was controlled by data saturation wherein an additional participant does not yield any new data and the sample size is then determined to be adequate [30].

### Entry to site

 Written permission was obtained from the Health Directorates of the Volta Region (Oti Region was not created yet) and the Nkwanta South Municipality to collect data in selected health facilities within their jurisdiction. Additionally, permission was obtained from the management of hospitals where some of the participants were recruited for the study. Written permission was also obtained from the Nkwanta South Municipal Assembly enabling the researchers to collect data from caregivers in households within the municipality. Assembly members and opinion leaders within the communities acted as ‘gatekeepers’ within their respective jurisdictions. Opinion leaders are individuals who wield influence within their communities and can sway the opinions of members of their communities. These individuals were contacted to facilitate smooth data gathering process. Being a patriarchal setting, the verbal permission of male household heads was necessary for the women to be allowed to grant interviews to the researchers. The interviewer, who is also one of the authors (FKN), explained the purpose of the study and the role of caregivers in the study to the heads of household who gave their consent for their spouses to be enrolled in the study.

The individual nurses were approached by the researcher following an introduction by the nurse managers and unit heads. The purpose of the study was explained to each of the nurses, and they all consented to take part in the study. Also, the nurses in the health facilities introduced the researcher to the caregivers who had visited the facilities with their children for healthcare services. The researcher subsequently explained the purpose of the study to each of the caregivers, and they all consented to be interviewed. Caregivers who were interviewed in their homes were identified by nurses who lived and worked in those communities with the assistance of the Community Health Volunteers. The researcher approached and invited these caregivers to take part in the study after he was introduced to the caregivers by the nurses.

### Data collection

Data collection was from January to March 2019. Face-to-face semi-structured interviews were conducted with each participant using an interview guide for each group. All interviews were conducted by field researcher (FKN), who is also one of the authors. The interviewer, the interviewee, and the gatekeeper agreed on a suitable date and time that ensured that the activities of the units and participants were not adversely affected by the interviews. The participants were selected as described above and were interviewed in a serene environment that ensured that they were relaxed and felt at ease in responding to the interview questions. Interviews with nurses and caregivers in the health facilities were conducted in open spaces under trees located on the premises of the health facilities to avoid interruptions and to also safeguard the privacy of participants. A few of the nurses were however interviewed in their offices alone after they had closed for the day. Interviews with caregivers in the communities were conducted either in a quiet corner of the house or outside the main compound of the house of the caregivers, depending on which one provided a serene atmosphere for caregivers to express themselves freely. Since most of the caregivers are farmers, the interviews in communities were conducted in the late afternoon or evening after caregivers had returned from their farms. Each participant was assigned a code that helped to associate the respondent with a particular group in the study while maintaining their anonymity.

The interviews were recorded using a digital recording device. All the interviews with the nurse participants and four of the caregiver participants were conducted in the English language while the remaining five caregiver participants’ interviews were conducted in the *Twi* language, a popular Ghanaian language spoken by the interviewer and most residents including the caregiver participants in the study area. The interviews lasted between 45 and 60 min and 30 min each for the nurses and caregivers respectively. After each interview, the audio recordings were played back for clarity and audibility, and for the participant to offer clarification when and where necessary. The recordings were then transferred onto the researcher’s laptop for storage and processing. Fieldnotes as well as the researcher’s reflections made during data collection were inserted into the interview transcripts before data analysis [30, 31].

### Data Analysis

Data gathering and data analysis were done simultaneously as is the practice in qualitative studies [37]. Some of the topics included in the interview guide for nurses were their experiences with the provision of child healthcare services and the main barriers they experienced in the provision of child healthcare services in the study area. For the caregivers, the topics included their experiences with and reasons for the utilisation of child healthcare services and the challenges they experienced in the process of utilising child healthcare services. Prior to the actual data collection phase, the researcher selected two participants who met the inclusion criteria and conducted two initial pilot interviews: one with a nurse at the children’s ward of one of the hospitals, and the other with a caregiver. The study applied the steps of qualitative data analysis outlined by Creswell [38] in the analysis of the data. After the interviews, the researchers engaged with the raw data by listening to audio recordings of interviews and renaming the audio files to reflect the participants’ codes. The audio recordings of interviews that were conducted in the English language were transcribed verbatim by the researcher. Those interviews that were not conducted in English were transcribed and translated into the English language by a language expert to ensure that no meaning was lost in the process. The data were catalogued according to the group of participants and prepared for analysis. The researchers immersed themselves in the data to make meaning of it and put down their general impression about the data [38]. Coding and organising of data into categories were done using Atlas.ti for Mac (version 8).

The data were coded by one of the authors (FKN) and an independent coder using the eight-step coding process described by Tesch [39]. The codes were generated inductively. According to this process, the researcher engaged with raw data to get a picture of the whole; here, ideas that came to mind were written down. By this initial engagement with the data, ideas that came to mind were noted as memos. The researcher then picked each semi-structured interview transcript and read through it carefully. After going through all the interview transcripts and fieldnotes, each interview was again read through carefully. At this stage, a list of the topics found in the collected data was developed. Open and *in vivo* codes were converted to appropriate axial codes by looking for appropriate words and phrases that captured these codes. Similar topics were clustered and formed into columns. Categories and themes were identified and matched with their appropriate descriptive topics. An independent coder who holds a PhD in nursing and is experienced in qualitative data coding was engaged to assist with the coding and to enable the triangulation of data analysis, as well as to ensure the trustworthiness of the data analysis process.

### Ethical considerations

 All methods were carried out in accordance with relevant guidelines and regulations. The study protocol was approved by the Nelson Mandela University’s Research Ethics Committee (reference number: H18-HEA-NUR-018) and the Ghana Health Service Research Ethics Review Committee (reference number: GHS-ERC014/11/18). Participants were selected according to the procedures described above. Written informed consent to indicate their willingness to participate in the study was obtained from each participant prior to the data collection. The contents of participant information sheet were verbally translated into *Twi* by the researcher to participants who could neither read nor comprehend English language. This was done in the presence of a witness who also signed the consent form to indicate that the translation as well as clarifications sought by the participants were done accurately. Also, verbal consent to publish the results was obtained from each participant before the start of the interviews. Participants were duly informed of the role they will play in the study and of their right to withdraw from the study at any time for any reason. Participants’ right to privacy was upheld as interviews were held at locations agreed upon with participants, as well as anonymising their responses to ensure confidentiality. The study adhered to all the requirements outlined in the Protection of Personal Information Act, 2013 [40].

### 2.6 Trustworthiness

The criteria of credibility, dependability, transferability, and confirmability were used to ensure trustworthiness in the current study [31]. To ensure credibility, the researchers consciously ensured that they went by the interview guide and stuck to the subject matter of the interview during the data collection process as part of reflexivity. Also, data from different groups of participants were triangulated, to capture various views regarding the socio-cultural barriers to the delivery and utilisation of child healthcare services in the Nkwanta South Municipality. One researcher and an independent coder coded the data, which contributed to ensuring the credibility of the data. By providing a thorough description of the entire research process, including in-depth description of setting, context, data collection and analysis, other researchers and readers can replicate it in similar settings or participants, thus upholding transferability. Again, by defining the inclusion criteria, the researchers made sure that only participants who were qualified by way of experience and location were recruited for the study. The researcher maintained a neutral point of view by allowing the data to speak for itself (inclusion of direct quotes from participants) to ensure confirmability. In addition, the data was transcribed verbatim, with direct quotations from participants included in the discussion and presentation of findings to ensure that the perspectives of the participants were adequately captured.

## Results

The study comprised of ten nurses who rendered child healthcare services and nine caregivers of children under-five who utilised the available child healthcare services within the Nkwanta South Municipality. The nurse participants were made up of six females and four males who ranged between 27 and 35 years of age. The nurse participants worked in different levels of healthcare delivery within the Primary Health Care system; that is, in the hospital, health centre, or CHPS compounds. At the CHPS level, the clinic nurse, designated as Community Health Officer, is the head of the healthcare team. The caregivers were all females who resided in different parts of the study area. The youngest caregiver was aged 23 years and the oldest was a 60-year-old grandmother taking care of her orphaned grandchildren. Three caregivers had no formal education; one attained basic education; four completed senior high school and one participant had attained tertiary education. The majority [5] of the caregivers were married, one each was widowed or single and two were co-habiting with the father of their youngest child. Seven of them were Christians, and one each was a Muslim and a Traditionalist.

The results revealed three main themes and six sub-themes as shown in Table 1 below:

**Table 1:** Themes and sub-themes that emerged

Themes	Sub-themes
1. Socio-cultural beliefs and practices	1.1 Cultural and spiritual beliefs about certain childhood diseases 1.2 Beliefs and practices affect child healthcare services 1.3 Male partner dominance
2. Language barrier	2.1 Challenges with language barrier 2.2 Negative impact of language barrier on nurse-caregiver relationship 2.3 Strategies to overcome language barrier
3. Reliance of caregivers on self-medication	3.1 Caregivers’ perspective 3.2 Nurses’ perspective

### Theme 1: socio-cultural beliefs and practices

 Socio-cultural beliefs and practices of caregivers about causation and management of certain childhood diseases and male dominance in decision making emerged as barriers to the utilisation of child healthcare services for children under five years of age. These beliefs and practices were found to be very common among caregivers and corroborated by nurses who provide child healthcare services in rural areas.

#### Sub-theme 1.1: cultural and spiritual belief about certain childhood diseases

Caregiver participants perceived disease conditions like convulsions in children to be caused by evil spirits and their management required the use of traditional medicine and not orthodox treatment. They believed that these diseases were caused by evil spirits transmitted by evil birds and other creatures.

*“Convulsions, usually happen when evil birds that fly in the sky, fly over the head of a child. That child will suffer convulsions and may die” (Caregiver 6).*

Caregivers described treatment modalities for these diseases perceived to be of spiritual origin:

*“They dig the roots of certain trees, peel the root and pound it into a mixture. You have to provide a fowl to be slaughtered as part of the process. You will be given some of the medicine to be given to the child then you will also be given some that after bathing the child you put it in fire and allow the child to inhale the smoke. You repeat this for three days if the child is male and four days if she is female. The convulsions will cease” (caregiver 6).*

According to the nurse participants, caregivers’ perceptions of certain diseases were deeply rooted in their lack of knowledge about the causation and management of those illnesses in children. These beliefs also were influenced by churches, prayer camps, and priests who discouraged caregivers from utilising child healthcare services.

*“There are some churches and prayer camps around who also tell them that their cases are spiritual…. even when they come [to the health facility], they still have a strong conviction that ‘this condition, this is what must be done for it to go’. So, whatever you are even telling them that the patient is suffering from; they will still be mentioning some other diseases that they think their child is suffering from” (Hosp. nurse 4).*

*“Another challenge we have is the spiritual homes around us. Instead of them encouraging the clients to come to the hospital, they keep them and brainwash them that their conditions are spiritual” (Hosp. nurse 6).*

*“When it comes to the management of certain hereditary conditions like sickle cell disease and anaemia, it becomes a problem. Because you are managing someone who thinks that sickle cell disease is as a result of someone giving* (infecting) *you* [with] *the disease.” (Hosp. nurse 5).*

#### Subtheme 1.2: beliefs and practices affect child healthcare services

Nurse participants noted that caregivers only turned to healthcare facilities after they had exhausted all the spiritual at-home illness management strategies available to them, without any relief. Caregivers however failed to disclose the remedies they had tried at home to healthcare workers, thus making the planning and care of their children quite difficult.

*“… for such a person, unless the sickness is very serious, and they have tried ‘things’ in the house which failed before they will bring the case here” (Clinic nurse 3).*

*“Because some still have a primitive mentality and they use local concoctions and what their grandparents and great grandparents believed. They still want to try it in the house and if it doesn’t work then they come* [to the hospital]. *… When they come, and you want to take all that history, they will be hiding, saying they did not do or give anything at home. But in actual fact, they have tried all their local remedies at home before coming” (Hosp. nurse 4).*

Nurse participants further indicated that caregivers adorned their children with objects of spiritual significance that interfered with nursing care delivery when the children are brought to the health facilities. Also, caregivers took their children on admission out of the hospital without permission to go and administer traditional and alternate medicine to the sick children, thus disrupting their children’s course of treatment. Other caregivers sneaked in these traditional and alternate medicines and applied them on the children at the blind side of the nurses.

*“But it is these chains they* (children) *have on their neck and waists, which are so tight if you ask them to remove it, they don’t want to, they think it is what is protecting the children” (Hosp. nurse 1).*

*“Sometimes they sneak out. They go with the child to the house and by the time they return they would have done it to the child or those that will come visiting from home will bring certain concoctions from the house and give it to them and they apply it on the child.” (Hosp. nurse 4).*

#### Subtheme 1.3: male partner dominance

 The dominant role of male partners of caregivers in decision making, as well as providing for the family was another dimension of the socio-cultural practices that negatively affected the utilisation of child healthcare services. Both nurse and caregiver participants described a patriarchal society where caregivers have very little say, if any, in decisions pertaining to the utilisation of child healthcare services when needed.

*“You know, this area, they respect their men a lot and they believe that every decision must be taken by the man. So, you the woman cannot take any decision on your own. And so, if the child is sick* (meanwhile the child is in the care of the mother), *the mother is waiting for her husband to come and tell her to go to the hospital before they come. And it takes a long time for them to take that decision” (Hosp. Nurse 7)*.

*“Males make most of the decisions. I think that is even one of the barriers to healthcare in this area. If the child is sick, and for the mother to bring the child to hospital, the father has to approve of it. I don’t know whether it is part of their tradition or culture or something like that” (Hosp. nurse 4).*

Caregivers often deferred decisions about child healthcare services to their husbands, out of reverence for men. One caregiver narrated how she had to comply with her husband’s orders to return home with their sick daughter who was referred to a regional hospital for advanced medical care and attention:

*“I am a woman; I can’t go contrary to my husband’s instructions. You are also a man (*referring to the interviewer*) and you know what I am talking about. As a woman, I have no power when it comes to decision making, so whatever my husband says is what I do. So, when he received the referral letter, he asked that we should take the child home… I had no option but to oblige to his instructions” (Caregiver 7).*

According to the nurses, caregivers required the permission of their husbands to bring their sick children to the hospital. However, the reluctance of male partners to make prompt decisions regarding the healthcare needs of their children and wives sometimes led to complications and poor treatment outcomes.

* “The fact is that, here, if the father agrees to whatever you say, that is the final. But if the fathers do not agree to it then it becomes a problem. (Hosp. nurse 4).*

*“At times if you ask them* (caregivers) *they say that the father is not around. He is in the bush* (farm)*…she calls the father to come before they can come* (to the hospital)*” (Hosp. nurse 2).*

*“Severe birth asphyxia is the leading cause of our admissions here. Sometimes too, consenting to Caesarean section, the woman will say ‘I’m waiting for my husband’. … So sometimes all these things make it difficult for the doctors and in the long run we end up receiving asphyxiated babies who are admitted at the neonatal unit” (Hosp. nurse 7).*

Some nurse participants also noted that male dominance thrived in these communities because the women lack economic empowerment. The women are dependent on their male partners to provide for the family, including settling their medical bills.

*“When your husband says there is no money, I don’t know how else they can raise money” (Clinic nurse 1)*.

*“When we go for home visits and we meet them* (caregivers) *at home, they complain that their husbands don’t give them money to come to the clinic…. Even when they come for Child Welfare Clinic and we see that the child is sick, and we inform them, they say the father of the child has not given them money” (Clinic nurse 3).*

The general belief in these communities is that fathers ‘own’ their children and, therefore, they must provide for the wellbeing and welfare of ‘their’ children.

*“The women, sometimes, they say that the child belongs to the man. So, he must take care of the child and everything. So, when the child is sick, everything is on the man. Whatever happens to the child, it is the man’s problem” (Clinic nurse 1).*

### Theme 2: language barrier

Language barrier emerged as another socio-cultural barrier that hampered the smooth delivery and utilisation of child healthcare services in the study area. Participants described the causes of this problem, its impact on the child healthcare delivery and utilisation, and how they muddled through this challenge.

#### Sub-theme 2.1: language barrier as a result of the multiplicity of languages

 According to the nurse and caregiver participants, language barrier was a challenge that they had to grapple with in their daily interactions. The lack of a common language was noted as a cause of confusion between nurses and caregivers which also hampered the smooth exchange of information. Caregivers described feeling incensed because they did not understand what the nurses were saying. Some of the participants explained this situation in the following quotes:

*“Most of them* [caregivers] *speak Konkomba, Chokossi, or Bassari; I am an Ewe and my Twi is not that okay… Because when I don’t understand what you are saying, I can’t give you what you want” (Clinic Nurse 1).*

*“…sometimes language barrier is a problem. Maybe what the person (*nurse*) is saying we don’t understand. It may be that they are not talking about us but since you may be the only person present at the time, you feel irritated” (Caregiver 8).*

The nurse participant attributed the cause of this challenge to the multiplicity of languages spoken in the study area coupled with the high level of illiteracy (hence unable to speak the English language) among the residents.

*“We have about fifteen languages here. Sometimes when you want to explain something to the relatives, they don’t understand, and they don’t get it… Because their level of education is low if they were much educated you could put it into writing. Because they are not educated it becomes very difficult.” (Hosp. Nurse 4).*

*“Sometimes some of the mothers don’t understand the [English] language” (Clinic Nurse 3)*.

#### Sub-theme 2.2: negative impact of language barrier on nurse-caregiver interactions

 Nurse participants acknowledged that language barrier affects the dissemination of vital information to caregivers, especially pertaining to home care after discharge. A nurse participant indicated that they withheld vital information from patients because of language barrier.

*“… especially if they are about to be discharged and you want to give them education before they leave, you end up not knowing what to tell them because they don’t understand, and you don’t know what to do…then we keep the education to ourselves and do our best. That is why some of them do not comply with treatment because education was not given because of language barrier”. (Hosp. Nurse 4)*

A nurse participant observed that caregivers sometimes did not cooperate with the nurses as they (caregivers) did not understand precisely what the nurses want to do to their children. Nurses became helpless in such instances.

*“So, let’s say you want to give a vaccine and you tell the woman that this vaccine protects against measles, they don’t even know [understand] what measles is. … And we don’t also know how to mention it in a language they can understand. So, I think language is key” (Clinic nurse 3)*.

#### Sub-theme 2.3: strategies to overcome language barrier

 To overcome the linguistic challenge, and facilitate communication between nurses and caregivers, the nurses relied on their colleagues or relatives of other patients who could speak the local languages to interpret the communication for them:

*“…for the local languages, we have nurses who can speak those languages. So, whenever there is a language barrier and your colleague who is there can speak that language, you just fall on him/her to discuss with the mother” (Hosp. Nurse 3).*

*“If it* (language barrier) *happens like that, we go to the ward and ask if any of the mothers here understand this language. So, sometimes the relatives of other patients come in to translate to the new one” (Hosp. Nurse 1).*

Nurses in the clinics relied on the community health volunteers in the clinics to interpret communication between them and caregivers when the need arose.

*“We have one of our volunteers at ‘Fulani Line’ (one of the communities), whenever we call, and he is around, he comes to do the interpretation for us” (Clinic Nurse 1).*

However, the nurse participants described experiencing several challenges while relying on other people to interpret information between them and the caregivers. Some of them narrated their experiences in the quotes that follow:

*“The time that you will take to call someone to come and do the interpretation for you to diagnose the patient takes a lot of time… It is the time spent on one client that is my problem” (Clinic Nurse 1).*

Attempts by relatives of caregivers to stay on and assist with interpretation of communication between healthcare workers and the caregivers led to congestion on the wards. One nurse at the hospital explained how attempts by nurses to decongest the ward by sending away families who wanted to stay with their relative (to help in the interpretation of communication) ended in an altercation between the nurses and the relatives of the caregiver.

*“They are Fulani people. The mother doesn’t understand Twi, she can speak only Fulani. So that day they came by 6pm and the whole family about ten were on the ward and insisted that they wanted to sleep on the ward. … We said, ‘give us only one person who understands Twi so that we can communicate through him and the mother will get the information’. That day was tough, it was a night shift. It was in the morning that people intervened, and they calmed down. They were trying to exchange words with the staff, that they want to stay (*to assist with communication)*” (Hosp. Nurse 2).*

### Theme 3: reliance of caregivers on self-medication

Data from the interviews with both caregiver and nurse participants revealed that the reliance on self-medication in the management of sick children is widespread in the study area.

#### Sub-theme 3.1: caregivers’ perspective

 Caregiver participants stated that they looked out for several indicators that would point to ill-health in their children before proceeding to administered medications at home. These indicators are usually in the form of deviations from the child’s normal level of activity or obvious signs of physiological changes in the child. The following quotes from some of the caregiver participants lend support in this regard:

*“In the house, the child was very weak, he doesn’t like food and at times he vomits, so I talked to my husband that ‘today our boy is not feeling well’” (Caregiver 4).*

*“But before I realise that he didn’t eat, he would have become very hot to touch and then convulse” (Caregiver 8).*

Caregiver participants further indicated that they relied on self-medication to manage ill-health in their children. These medications were either leftovers from previous health facility visits or from chemical shops located in the communities where caregivers reside. They administered these treatments with the hope that it could improve the conditions of their sick children.

*“The first time we came to the hospital with cough, they gave us some cough syrup; so, I still have some of the syrup. When it happened, I gave him some and said let me try this in the house and see (Caregiver 2).*

*“When my husband is not there to carry us to Ofosu [*health centre*], I go to the drug store [to get medication]” (Caregiver 9)*.

Caregivers expressed varied opinions regarding the potency of these home remedies. They intimated that the remedies worked sometimes but failed in other instances. They took their sick children to the healthcare facilities when these home remedies failed to produce the expected results. These quotes from some of the participants lend credence to this statement:

*“At times it works and at times too you will have to go to the hospital before the sickness will go. Like the beginning of the sickness, the first day it begins, you may say that this one when I go to the drugstore, she will be better so let me go to the drugstore first but when you go there and you see that things are getting worse, it is then that you will rush to the hospital” (Caregiver 5).*

*“…They (drugstore) gave me ORS, anti-malarial drugs and haematinics. When I gave them to the child, he became well” (Caregiver 9)*.

*“If after some time there is no visible improvement, I know whatever is wrong with him is beyond Paracetamol syrup and I should take him to the hospital” (Caregiver 8).*

#### Sub-theme 3.2: nurses’ perspective

Nurse participants were of the view that reliance of caregivers on self-medication was one of the reasons why caregivers delayed in bringing their sick children to the healthcare facilities:

*“Well, generally, they don’t like coming to the facility often when the child is sick; they don’t come on time. They wait for some days hoping that the child will be well, buying some stuffs like medicine from town, self-medication, and stuff. When the sickness gets serious before they’ll come” (Clinic nurse 2).*

The nurse participants were not enthused about caregivers’ trust in these over-the-counter drugs and the treatment of children at home without accurate diagnoses. Besides, the nurse participants felt that caregivers were unable to accurately describe the type of self-medication that was administered to the sick children prior to visiting the hospital.

*“And sometimes too, these over-the-counter drugs, they believe in it and so they go in for them not really knowing what condition the child is suffering from. All they know is my child is sick and I need to get a drug for my child, so they go and buy them… When you ask them what they gave to their child, they tell you ‘tidar’, paracetamol syrup. Meanwhile, it’s not paracetamol syrup or ‘tidar’ the child needs at that moment” (Hosp. nurse 7).*

## Discussion

The results of the study revealed the socio-cultural barriers that impede the utilisation of child healthcare services in rural communities in Ghana. The findings related to cultural beliefs and practices about certain childhood illnesses are congruent with those of previous studies, which reported that illnesses perceived to have a supernatural aetiology were often managed using traditional medicine and other home remedies [41–43]. The three delays model recognises sociocultural barriers as contributing to delay in the decision to seek care and its attendant undesirable health outcomes [12]. Nurses perceived that caregivers’ decision making about the causes and treatment of children’s illnesses was based on their lack of knowledge of the causation of certain illnesses and influenced by the activities of spiritualists, churches, and prayer camps. Perceptions about the causes of illnesses to a large extent determine the care seeking path for such ailments as illnesses believed to be spiritual in origin often involved the use of plant parts and spiritual treatments, and prayers [43, 44]. Cultural and religious beliefs have been found to be a hindrance to treatment seeking for children with certain conditions as caregivers tend to prefer alternative forms of treatment including traditional medicine, prayers, and the use of holy objects as the first point of treatment when the cause of the illness was thought to be spiritual [45, 46]. Caregivers resorting to traditional medicine delayed the prompt utilisation of child healthcare services and the smooth delivery of care to sick children as caregivers failed to disclose treatments that they had administered to the child prior to coming to the hospital. Also, by taking the sick child out of the ward to go and administer traditional medicine preparations to them, the smooth delivery of nursing care is disrupted. Furthermore, the adornment of children with certain objects believed to offer them protection caused interference to smooth rendering of nursing care. These findings support several previous studies that have reported the use of traditional, complementary, and alternative medicine, especially among rural dwellers in Sub-Saharan Africa, and reasons why sick children may present late to healthcare facilities [47–52]. Early and prompt utilisation of child healthcare services is the best way to prevent complications and guarantee a good outcome. Hence these beliefs and practices of caregivers could contribute to poor child healthcare outcomes.

The dominant role of males in the household decision making was also found to be a hindrance to utilisation of child healthcare services when needed. It was noted that men wield the decision-making authority because they are considered the heads and breadwinners of their households and their failure to make prompt decisions or grant permission for the utilisation of child healthcare services often led to complications and poor treatment outcomes. These findings are congruent with previous studies where male partners determined the healthcare seeking path for sick children and made decisions on whether and when to use orthodox healthcare as they were responsible for footing the bill of the child who required said healthcare services [25, 42, 53–56]. In a typical rural Ghanaian setting, men are generally seen to be the heads of households and are responsible for the provision of economic support for the members of their households [57]. The lack of economic empowerment of women, and the patriarchal nature of the study setting, where males are considered as owners of the children and must provide for them accounted for this situation. In addition to the direct cost, caregivers incur additional costs such as cost of transportation and feeding when they have to access child healthcare services outside their communities. Caregivers in the Nkwanta South Municipality mostly assist their husbands who engage in peasant agricultural activities and hence do not earn enough to be able to afford the total cost of child healthcare services [36]. Financial constraints have been documented as contributing to mothers’ reluctance to utilise child healthcare services in Ghana and Malawi [21, 47]. According to Manda-Taylor et al. [58], the failure of husbands to foot healthcare bills hindered women’s ability to utilise child healthcare services in Malawi, as the cost implications of child healthcare services are often beyond the capabilities of caregivers, who mostly do not earn enough.

 Language barrier between nurses and caregivers resulted from the multiplicity of local languages spoken in the study area which reduced the chances of nurses, and caregivers being able to interact effectively in a common language. The Nkwanta South Municipality is one of the most ethnically diverse districts in Ghana, with about a quarter of all Ghanaian languages being spoken in this area [35]. While the majority of the residents speak and understand the local languages, most of the health workers who are not natives of the area could neither speak nor understand these local languages. Though *Twi* is a widely spoken language in this part of the country, the caregivers, particularly those from the rural communities, are not very fluent in it. Besides, nurses who come from parts of the country where *Twi* is not predominantly spoken may also not be fluent in it. Additionally, low literacy level in the area means that the majority of caregivers are unable to express themselves in English language; thus, making it impossible for them to interact with healthcare workers in English language. It is estimated that over 40% of residents in the Nkwanta South Municipality never attended school and over 80% of all those with some education did not go beyond basic education [34, 36].

Language barrier posed a challenge to the delivery and utilisation of child healthcare services in the study area as it caused delays, poor communication of health information, a hindrance to patient education, and reduced involvement of caregivers in the delivery of child healthcare services. These findings are consistent with previous studies in Ghana [59–61]. In Switzerland, the Netherlands, and the US, language barriers made healthcare delivery and utilisation difficult, especially in emergencies [62–64]. Language as the medium of verbal communication facilitates understanding and sharing of vital information among healthcare workers and patients and their relatives. It is even more crucial in child healthcare service delivery where the caregiver is the main decision maker on behalf of the child. The current system of selection and posting of newly trained nurses by the Ministry of Health does not consider the background or competence of nurses in local languages of the communities where they are posted or the context within which the nurses will work, whether in a hospital or CHPS compound. The interactions between nurses and caregivers became significantly impaired and characterised a feeling of lack of respect in the face of a language barrier, thus impeding the smooth delivery and utilisation of child healthcare services. Language thus affected respectful care since caregivers felt irritated when they did not understand what the nurses were saying. Respectful care is a fundamental human right and necessary in promoting maternal and child healthcare services utilisation [65]. It forms a central component of family-centred care which focuses on the positive aspects of care that patients and their families deserve [66, 67]. Respect and dignity, as well as effective communication form vital components of quality care in a health system [65]. Whereas nurses held back vital information from caregivers because of the language barrier, caregivers also felt inadequately cared for by the nurses. Ganle, et al. [59] reported in their study that language barrier is often misconstrued by caregivers as maltreatment by health workers. Similarly, in Ghana, Ohene, et al. [68] found that caregivers who could not comprehend nurses’ instructions were perceived as difficult by nurses. To overcome the challenge of language differences, the nurses in the current study adopted strategies including relying on colleague nurses and patient relatives as interpreters. Relying on third parties for interpretation of communication between nurses and caregivers however, posed challenges including delays in the delivery of essential child healthcare services. These findings support several previous studies [56, 62, 69, 70].

Self-medication as first-line treatment of sick children was found to be common among caregivers. The caregivers self-diagnosed and self-medicated their sick children. The findings above confirm those of previous studies which noted that the widespread use of self-medication to treat sick children in Sub-Saharan Africa [71, 72]. Self-diagnoses of ill-health in children could prove fatal because caregivers might miss danger signs in their children [42, 58, 73]. Ameh, et al. [72], reported that, in the Kassena-Nankana district in Ghana, caregivers resorted to purchasing medication from drugstores because these facilities were affordable and readily available within their communities. Self-medication of sick children could lead to delays as these treatments offered temporary relief of symptoms and could mask serious conditions. Caregivers expressed mixed reactions about the effectiveness of self-medication in treating sick children and often reverted to healthcare facilities when they did not attain the desired results. This practice contributed to delays in seeking child healthcare services. Previous studies have found that self-medication and the use of traditional medicine accounted for delays in seeking treatment for sick children [21, 42, 74]. Caregivers reportedly reverted to orthodox treatment for their sick children mostly after their home remedies failed [73–76]. Late reporting to healthcare facilities to seek appropriate treatment could lead to poor treatment outcomes. Efforts at improving child healthcare outcomes cannot yield the desired results if caregivers do not report for treatment early enough but continue to rely on home remedies until their children’s conditions deteriorate.

 The study uncovered several socio-cultural barriers experienced by both caregivers and nurses regarding the delivery and utilisation of child healthcare services in a rural setting. A qualitative research approach ensured in-depth exploration of the socio-cultural barriers from the perspectives of the study participants. By exploring the perspectives of both caregivers and nurses at various levels of the primary healthcare system, the study generated rich data that enhanced the understanding of the socio-cultural barriers to child healthcare delivery and utilisation in a rural setting.

However, the qualitative nature of the study meant that the contribution of these barriers and the extent to which they affected the delivery and utilisation of child healthcare services could not be examined. Also, researchers concede the data was collected from nurses and caregivers in only one rural district. The perspectives of health service managers, opinion leaders, and male partners of caregivers may be explored by future studies to advance the discourse on the topic.

## Conclusions

It is evident from the findings of the study that nurses and caregivers experienced several socio-cultural barriers to the prompt and consistent utilisation of child healthcare services in rural areas. These socio-cultural barriers either delayed care seeking by caregivers for their sick children or interfered with the smooth and prompt delivery of needed child healthcare services by nurses which are essential in ensuring the proper growth and development of children. Also, some of the barriers negatively affected the interaction between nurses and caregivers with the tendency to affect subsequent child healthcare service utilisation. It is important to institute measures to overcome these barriers and facilitate the early and consistent utilisation of child healthcare services especially in rural areas.

Thus, it is recommended that strategies to facilitate the delivery and utilisation of child healthcare should address these barriers in their totality. Nurses should foster close collaboration with caregivers and community leaders to address these socio-cultural barriers. Health education by volunteers and community health officers to caregivers and their families should be intensified with more emphasis placed on the need to promptly report all illnesses in children to the health facility. The education should further emphasise on the negative impact of certain socio-cultural beliefs, and practices on child health. This will avert any adverse outcomes caused by delays and non-utilisation of child healthcare services. The Ministry of Health should consider fluency of nurses in specific local languages during postings of nurses to reduce the challenges of language barriers between nurses and caregivers. Discussions should be held with community leaders on empowering and supporting women to make quick decisions for their health and that of their children to facilitate the prompt utilisation of child healthcare services. The Municipal Health Directorate should engage with the spiritual leaders in the area to educate them on the essence of prompt utilisation of child healthcare services.

## Data Availability

The datasets used and/or analysed during the current study are not publicly available due to the study’s ethical requirements to ensure respondents’ anonymity in reporting and confidentiality in participating in the study; but are available from the corresponding author on reasonable request.
